# Simulation of the Keureuto Dam collapse disaster based on flood distribution

**DOI:** 10.4102/jamba.v18i1.2045

**Published:** 2026-08-18

**Authors:** Wesli Wesli, Fadhliani Fadhliani, Nanda S. Ersa

**Affiliations:** 1 Department of Civil Engineering, Faculty of Engineering, Universitas Malikussaleh, Lhokseumawe, Indonesia

**Keywords:** disaster risk mitigation; dam breach, hydrological modelling, flood simulation, HEC-RAS, Keureuto Dam

## Abstract

Dams play a vital role in supporting agricultural productivity, water supply and flood control in Indonesia. However, their structural failure poses serious threats to human safety and regional development. This study integrates hydrological and hydraulic modelling as part of a comprehensive disaster risk mitigation strategy, focusing on the Keureuto Dam in North Aceh, Indonesia. The dam, a 74-m-high earthfill structure with a total storage capacity of 215.94 million m^3^, presents significant downstream flood risk in the event of structural collapse disaster risk mitigation. Using the HEC-RAS 5.0.7 model, a dam breach scenario was simulated under overtopping conditions based on Froehlich’s empirical equations. The simulation results revealed a maximum flood depth of 19.577 m, a peak flow velocity of 1.387 m/s and an inundation area of approximately 49.91 km^2^ affecting 84 villages across four sub-districts.

**Contribution:**

Beyond quantifying flood characteristics, this study emphasises a decision-support tool for disaster preparedness, enabling hazard mapping, evacuation planning and the development of early warning systems.

## Introduction

Constructing dams for water storage is an effective solution to meet human needs. However, despite their benefits, dams also pose significant risks if failure occurs, potentially causing catastrophic downstream flooding and major losses within a short time (Coombs 2018; Srivastava & Sahoo 2023). According to Government Regulation of the Republic of Indonesia No. 37 of 2010, a dam is a structure built to retain and store water, including mining waste or sediment, forming a reservoir. Dams are large-scale hydraulic structures designed to store water and support various human needs.

To understand the distribution and movement of water on global, watershed and terrestrial scales, studying the hydrologic cycle is essential (Chow, Maidment & Mays 1988; Zeng & Chu 2021). This section discusses the definition, illustration and processes involved in the hydrologic cycle.

Dam safety simulations are important for flood risk mitigation and emergency planning, but deterministic peak flow estimates have limitations under uncertain conditions (Bilali et al. 2022; Timoshenko 2025). The dam failure scenario was simulated using the HEC-RAS software, considering potential failure mechanisms such as overtopping and piping (Tessema, Gebremedhn & Getahun 2024; Waryani, Sachro & Edhisono 2025).

Disaster risk assessment includes hazard identification, risk analysis and evaluation to determine potential impacts and support effective mitigation and monitoring measures (Hassel & Cedergren 2021; Zuccaro, Leone & Martucci 2020).

Dams are considered critical infrastructure because their failure can cause catastrophic impacts. Dam risk is commonly defined by the interaction of hazard, vulnerability and exposure. Historical failures show that dam disasters usually result from combined natural, structural and operational factors (Wiguna et al. 2022). A representative case of dam disaster risk is the failure of the Situ Gintung Dam in 2009 in Indonesia (Sujono 2012), which represents a typical overtopping and structural failure event. Extreme rainfall exceeded reservoir capacity, while the ageing embankment lacked sufficient resilience. Key risk factors included hydrological hazard, structural vulnerability and high downstream exposure, resulting in major loss of life and severe residential damage (Nabilah et al. 2020).

This study is about disaster risk mitigation, using hydrological modelling. The analysis was conducted in accordance with the Emergency Action Plan (EAP) guidelines established by the International Committee on Large Dams (ICOLD). The analyses are crucial for identifying potential flood-risk zones and developing effective early warning systems for downstream areas. The flood inundation and dam failure simulations were carried out using HEC-RAS version 5.0.7 (Ansori 2021).

Dam failures pose major risks to downstream populations, making loss-of-life assessments essential for emergency response and risk management (Jibhakate, Timbadiya & Patel 2024). Population distribution varies spatially and temporally, while warning timeliness and public response strongly influence evacuation success (Peng, Zhang & Sayama 2024).

Despite their benefits, dams can pose serious risks when failure occurs, potentially causing catastrophic flooding, loss of life and extensive downstream damage in a short time (Li et al. 2023). Dam failure floods can cause severe structural damage and loss of life. The interaction between flood dynamics and building collapse also significantly affects flood behaviour and risk assessment (Maranzoni, D’Oria & Rizzo 2024; Song et al. 2024; Zhou et al. 2022).

The Keureuto Dam, situated in Blang Pante Village, Paya Bakong District, North Aceh Regency, has a height of 74 m and a total storage capacity of 215.94 million cubic metres. To evaluate the potential consequences of a dam failure, it is essential to conduct a probabilistic analysis of collapse scenarios (Alief, Khairullah & Fadhliani 2024; Cleary et al. 2015; Pratama et al. 2024). Such an assessment is particularly critical considering the dam’s considerable height and storage volume, as well as the presence of populated areas in the downstream region (Mudita & Sherly 2023).

To perform this simulation, the HEC-RAS 5.0.7 software developed by the U.S. Army Corps of Engineers was utilised. This programme enables two-dimensional (2D) modelling of dam breach scenarios to simulate the resulting flood propagation, allowing for the assessment of flood extent and depth. Understanding the downstream inundation pattern of the Keureuto Dam provides valuable insights for developing effective emergency response plans aimed at protecting lives and property, as well as identifying the most vulnerable areas for evacuation (Beza, Fikre & Moshe 2023; Brunner 2010; Peng et al. 2014; Shrestha, Kafle & Bhattarai 2025).

This study aims to fill the gap in flood distribution and flood depth through a site-specific simulation model to predict the impact of the Keureuto Dam collapse using a hydrodynamics-based flood distribution approach, identify inundation zones, depth, velocity and flood arrival time in various dam collapse scenarios, and develop a flood hazard map as a scientific basis for emergency response planning and risk mitigation for downstream communities.

## Research methods and design

According to Standard National Indonesia SNI M-18-1989-F (1989), a flood is defined as a relatively high water discharge that exceeds the capacity of a river channel. A flood occurs when the flow of water exceeds the capacity of a river channel or embankment, causing water to overflow onto adjacent areas and disrupt human activities (Chow et al. 1988; Subramanya 2021; Zhao et al. 2025). The area along the left or right banks of a river that becomes inundated during a flood is referred to as the floodplain (Government of the Republic of Indonesia 2011).

A design flood area is a zone that can safely accommodate flood discharge for a specific return period. It is determined through hydrological analysis using parameters such as peak discharge, flood volume and flood hydrograph.

### Study area

**FIGURE 1:**
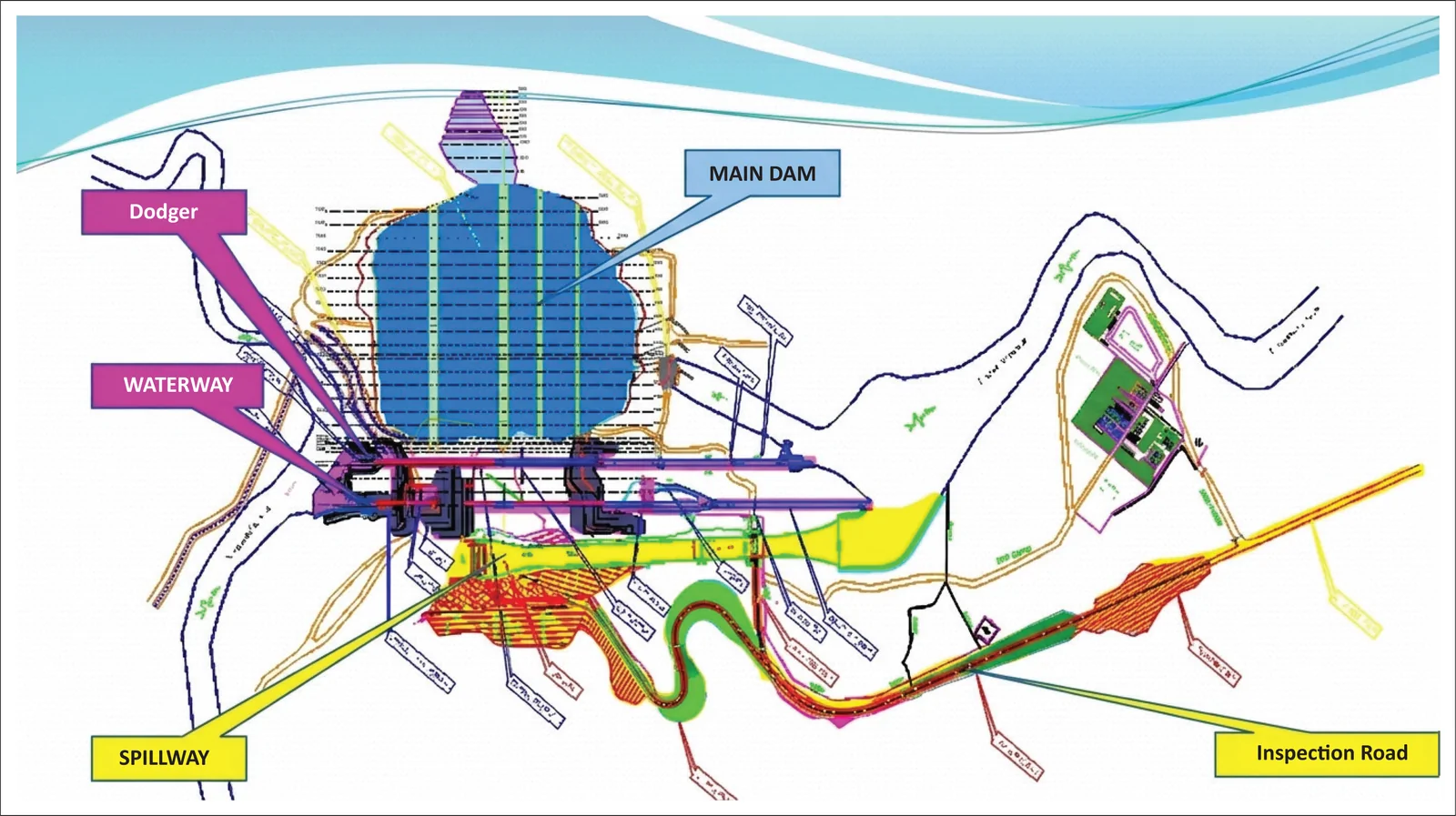
The situation map of the Keureuto Dam.

The Keureuto Dam is located in Blang Pante Village (population 2.145 people and 530 households), Paya Bakong District (population 3.210 people and 570 households), North Aceh Regency, Indonesia (Badan Pusat Statistik Kabupaten Aceh Utara n.d.) (Figure 1). The reservoir extends across several nearby villages, including Plu Pakam (population 1.820 people and 450 households) and Makarti (population 2.055 people and 510 households) in Tanah Luas District, and Rusip (population 257 people; 73 households) and Tembolon (population 234 people; 69 households) in Bandar District, Bener Meriah Regency (Badan Pusat Statistik Kabupaten Bener Meriah n.d.). Geographically, the area lies between 05°01’57.58” N – 04°49’52.97” N and 97°16’19.37” E – 96°58’24.32” E (Variadi et al. 2024). The watershed covers 69.18 km^2^ with a river length of 31.19 km, an inundation area of 896.39 ha, an average annual rainfall of 1619.58 mm and an average discharge of 13.53 m^3^/s. The situation map of the Keureuto Dam is shown in Figure 1.

The Keureuto Dam is an embankment-type structure with a height of 74 m and a crest length of 386 m. The total storage capacity is 215.94 million m^3^, divided into effective (167.22 million m^3^) and dead storage (33.95 million m^3^). The dam crest elevation is +107.51 m, the normal water level (NWL) +101.51 m and the flood water level +103.86 m. Additional specifications include a 358.8 m evacuation tunnel and a 372 m waterway tunnel.

The inflow discharges corresponding to return periods of 25 years, 1000 years and the probable maximum flood (PMF) are 707.78 m^3^/s, 1039.77 m^3^/s and 2680.88 m^3^/s, respectively. The sediment storage level is at +69.18 m, the low water level at +73.43 m, the normal water level at +101.51 m, the flood water level at +103.86 m and the flood control storage level at +98.01 m. The inundation area is 896.39 ha, with a total storage capacity of 215 million m^3^ (Balai Wilayah Sungai Sumatera 2022).

**FIGURE 2:**
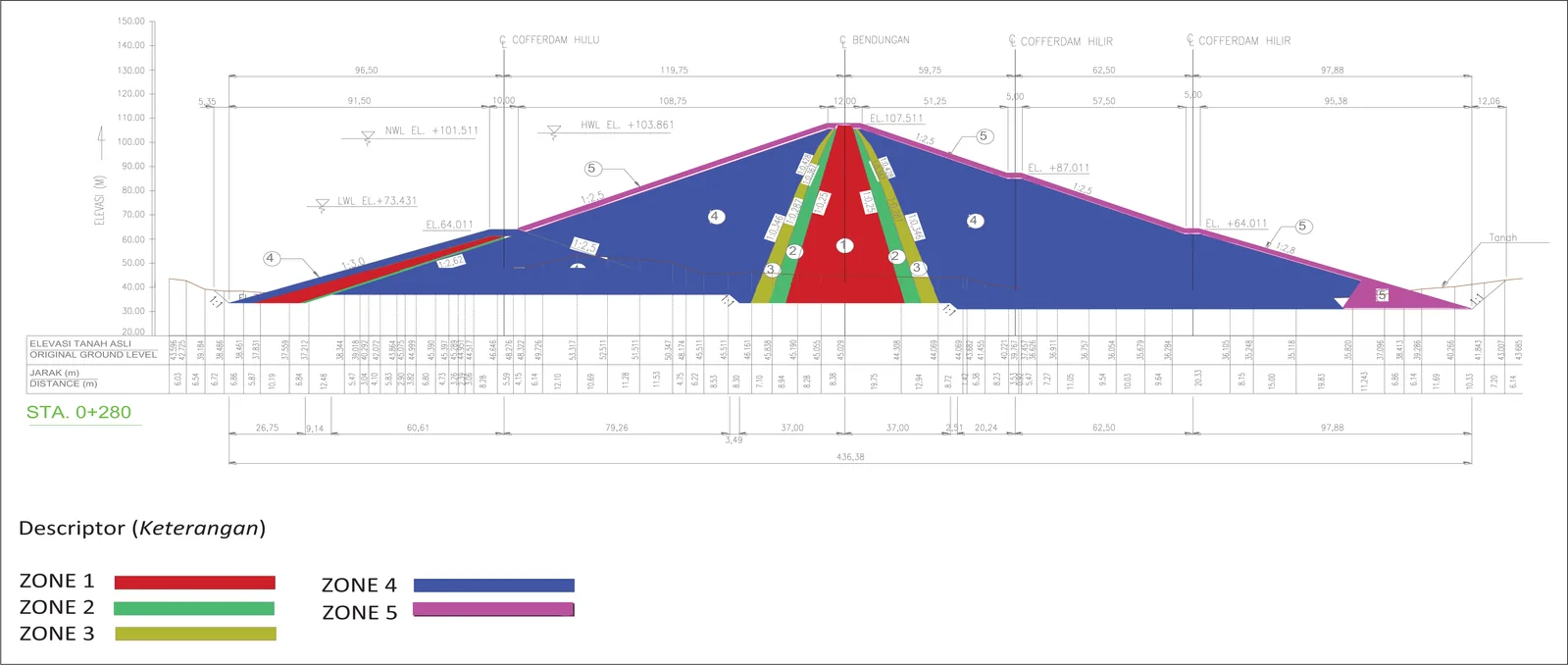
Technical data for the Keureuto Dam.

The technical specifications of the dam are as follows: It is a zonal-type embankment dam with a main dam height of 74 m and a total crest length of 386 m. The total storage capacity is 215.94 million m^3^, comprising an effective storage capacity of 167.22 million m^3^ and a dead storage capacity of 33.95 million m^3^. The reservoir covers an area of 896.39 ha at the normal water level (NWL). The dam crest elevation is +107.51 m, the intake elevation is +69.10 m, the spillway elevation is +101.511 m, the evacuation tunnel has a length of 358.80 m, and the waterway tunnel has a length of 372.00 m (Balai Wilayah Sungai Sumatera I 2024). The complete technical specifications of the Keureuto Dam are presented in Figure 2.

### Disaster risk reduction

The basic concept of disaster risk reduction (DRR) encompasses four main components: Risk identification and assessment, which involves analysing hazards, vulnerabilities, capacities and community exposure to disaster threats; mitigation and prevention, which focus on reducing the potential impacts of disasters through spatial planning, the development of disaster-resilient infrastructure and environmental conservation; preparedness and early warning, which include information systems, community training and technology-based early warning mechanisms; and recovery and rehabilitation, which emphasise the principle of *Build Back Better* to ensure communities become more resilient after disasters (Seddiky, Giggins & Gajendran 2020).

Acceptable risk is the level of disaster risk considered tolerable by society, balancing safety and practicality. It varies according to social, economic, political, cultural, technical and environmental conditions, and guides engineering design, policies and disaster risk reduction measures to keep potential impacts within manageable limits (UN Office for Disaster Risk Reduction 2024).

The United Nations Office for Disaster Risk Reduction (UNDRR) defines DRR as a systematic effort to identify, assess and reduce disaster risks. This approach focuses not only on emergency response, but also on prevention and mitigation efforts before a disaster occurs (ed. Brears 2022). Disaster risk reduction is a systematic approach to identifying, assessing and reducing disaster risks through mitigation, preparedness and community resilience strategies.

### Hydrological data

The rainfall data used in this study were obtained from the Meteorology, Climatology and Geophysics Agency (BMKG) North Aceh meteorological observation station located in and around the river basin (Table 1). The annual maximum daily rainfall data over a 20-year period were used for hydrological frequency analysis and PMF estimation.

**TABLE 1:** Annual maximum daily rainfall.

Number	Year	Annual maximum daily rainfall (mm)
1	2005	112
2	2006	125
3	2007	118
4	2008	136
5	2009	142
6	2010	155
7	2011	149
8	2012	161
9	2013	173
10	2014	168
11	2015	182
12	2016	176
13	2017	189
14	2018	194
15	2019	201
16	2020	214
17	2021	208
18	2022	223
19	2023	231
20	2024	245

mm, millimetres.

The statistical properties of the rainfall series summarised the number of data as 20 years, mean rainfall 176.1 mm, maximum rainfall 245 mm, minimum rainfall 112 mm, standard deviation 38.5 mm and coefficient of variation 0.22. The rainfall data were subsequently analysed using the Log Pearson Type III distribution to estimate design rainfall for various return periods.

In general, hydrological analysis is a fundamental component of the hydraulic structure design process. Subsequent analyses and design decisions depend heavily on the data and information derived from the hydrological study. The Nakayasu synthetic unit hydrograph method was applied to derive the probable maximum precipitation (PMP) and probable maximum flood (PMF) using the formula (Hershfield 1961) (Equation 1):


$$\text{Xm}=\bar{\text{x}}\text{P}+\text{Km}\times \text{Sp}$$ [Eqn 1]

where Xm is the maximum rainfall value or PMP; $\bar{\text{x}}$ P is the average of the *n*-year maximum rainfall data series; Km is the function value of the rainfall duration and the average annual maximum daily rainfall; and Sp is the standard deviation of the *n*-year maximum daily rainfall data.

Based on the probabilistic method, frequency analysis is conducted to determine the magnitude of the design rainfall. A distribution equality test was conducted to determine whether the results of the frequency analysis were acceptable. The distribution equality test was conducted using probability values of 1% and 5%. In this study, both distributions were used to measure the uniformity of the Chi-square and Smirnov–Kolmogorov distributions. The Hearfield method was used to calculate the maximum rainfall value (PMP) and obtain the design flood discharge (PMF) (Back & Bonfante 2021). Table 2 shows the results of the planned flood discharge calculations.

**TABLE 2:** Maximum design rainfall.

No	*T*	Planned rainfall (*Xt*) (mm)
1	2	132.9767
2	5	165.4302
3	10	180.1281
4	25	194.1915
5	50	202.4293
6	100	209.2978
7	PMF	5082.071

PMF, probable maximum flood; mm, millimetre.

Manning’s roughness coefficients were assigned based on land-use classifications and established hydraulic references, including Ven Te Chow (1988) and the United States Army Corps of Engineers guidelines. Spatial variation in roughness values was incorporated to represent differences in flow resistance associated with each land-cover type (Table 3).

**TABLE 3:** Manning’s coefficient (*n*).

Land use category	Manning’s coefficient (*n*)
Main river channel	0.030–0.035
Rocky channel	0.040
Cultivated land	0.045
Shrubland and low vegetation	0.050
Dense forest	0.080–0.120
Residential areas	0.060–0.100
Roads and open areas	0.015–0.020

Higher Manning’s coefficients were assigned to forested and residential areas to account for increased hydraulic resistance caused by dense vegetation, buildings and infrastructure. In contrast, lower coefficients were applied to river channels and open areas characterised by relatively smooth surfaces.

A sensitivity analysis was conducted to evaluate the influence of roughness uncertainty on model outputs. The results indicate that a ± 10% variation in Manning’s coefficient produced changes of approximately 3% – 8% in the maximum inundation depth, with the most pronounced effects occurring in low-gradient floodplain areas.

### Dam failure

In sediment failure analysis, the most uncertain parameters include fracture length, size, shape, position and formation time. The empirical equation developed by Froehlich, as documented in the *HEC-RAS User’s Manual*, has been widely applied in various dam safety assessments and breach analyses (Froehlich 1995).

In 2008, Froehlich conducted a study on 74 dam breach cases involving various types of embankments, including those with impervious (clay) cores and rockfill cores. The objective of the study was to develop empirical equations (Equation 2 and Equation 3) for estimating the average breach width, side slopes and failure time (Froehlich 2008).


$$\bar{B}=0.27K_{o}V_{w}^{0.32}H_{b}^{0.04}$$ [Eqn 2]

$$t_{f}=\sqrt{\frac{H_{w}}{gH_{b}^{2}}}$$ [Eqn 3]

where B is the average fracture width (m); *t_f_* is the fracture formation time (second); Vw is the volume at failure (m^3^); hb is the final fracture height (m); and Ko is a constant (1.3 for overtopping and 1.0 for piping).

Most dam failures result from a combination of factors, including overtopping, piping and foundation failure, each of which contributes to structural damage of the dam (Chen et al. 2023; Zhou et al. 2022). However, in the context of this study, particular attention was given to overflow as the primary cause of dam failure. This focus was chosen because HEC-RAS version 5.0.7, the analytical software employed in this study, is capable of simulating dam failure mechanisms related only to piping and overflow, but not other types of failure. Dam failures frequently occur due to overflow, which happens when water overtops the dam crest as the upstream inflow exceeds the dam’s capacity to manage extreme flood discharges. Moreover, each sediment transport function is highly dependent on site-specific geometry, hydraulic conditions and sediment characteristics, making it unsuitable for generalisation to other contexts (Basri et al. 2020). Furthermore, piping the flow of water through gaps or voids in the dam structure or foundation is a significant cause of dam failure, particularly when sedimentation compromises structural stability.

Model consistency verification was conducted by comparing the hydraulic behaviour of the simulation results with previously published dam-break studies. The inundation distribution pattern, which follows river corridors and low-topographic areas, also demonstrates agreement with similar studies using the 2D HEC-RAS model. This consistency in hydraulic behaviour indicates that the model configuration is capable of realistically representing flood-wave propagation and can therefore be used for dam-break flood hazard analysis. The results of the consistency verification are presented in Table 4.

**TABLE 4:** Model consistency verification.

Parameter	This study	Malpasset Dam (France)	Banqiao Dam (China)	Situ Gintung Dam (Indonesia)	Hydraulic behaviour consistency
Maximum flood depth	19.577 m	± 10 m – 20 m	> 20 m near the dam area	± 5 m – 15 m	Still within the range reported in previous studies
Inundation area	49.91 km^2^	Tens of km^2^	> 50 km^2^	Several km^2^	Shows a comparable inundation pattern
Flood propagation pattern	Rapid upstream flow, weakening downstream	Rapid wave propagation in steep valleys	Large flood waves spreading widely downstream	Inundation following the river corridor	Consistent with unsteady flow theory
Flood distribution	Following river channels and low-lying topography	Following the river valleys	Spreading across lowland areas	Following drainage networks	Realistic hydraulic pattern
Flow velocity	High near the dam	Very high in the initial breach zone	High due to large release volume	High in residential areas near the embankment	Consistent with energy dissipation characteristics
Numerical model	2D HEC-RAS	2D hydrodynamic model	Flood propagation model	2D hydraulic simulation	Comparable modelling approach

HEC-RAS, Hydrologic Engineering Center’s River Analysis System; m, metres; km, kilometre; 2D, two-dimensional.

Moreover, the characteristics of the downstream flood hydrograph can be influenced by various sediment-related failure parameters. The dam breach was modelled in HEC-RAS 5.0.7 using the 2D unsteady flow module. Inputs included dam geometry, reservoir elevation, breach side slopes and Manning’s *n* roughness (Dam Failure Parameters, Water Overflow Scenario as shown in Table 5).

**TABLE 5:** Dam failure parameters (overtopping scenario).

Number	Parameter	Value
1	Breach width (m)	196.83
2	Collapse time (h)	2.67
3	Collapse elevation (m)	111
4	Crest elevation (m)	111
5	Base elevation (m)	37
6	Flood water level (m)	111
7	Breach side slope	01:01
8	Dam coefficient	2.6

m, metres; h, hours.

**FIGURE 3:**
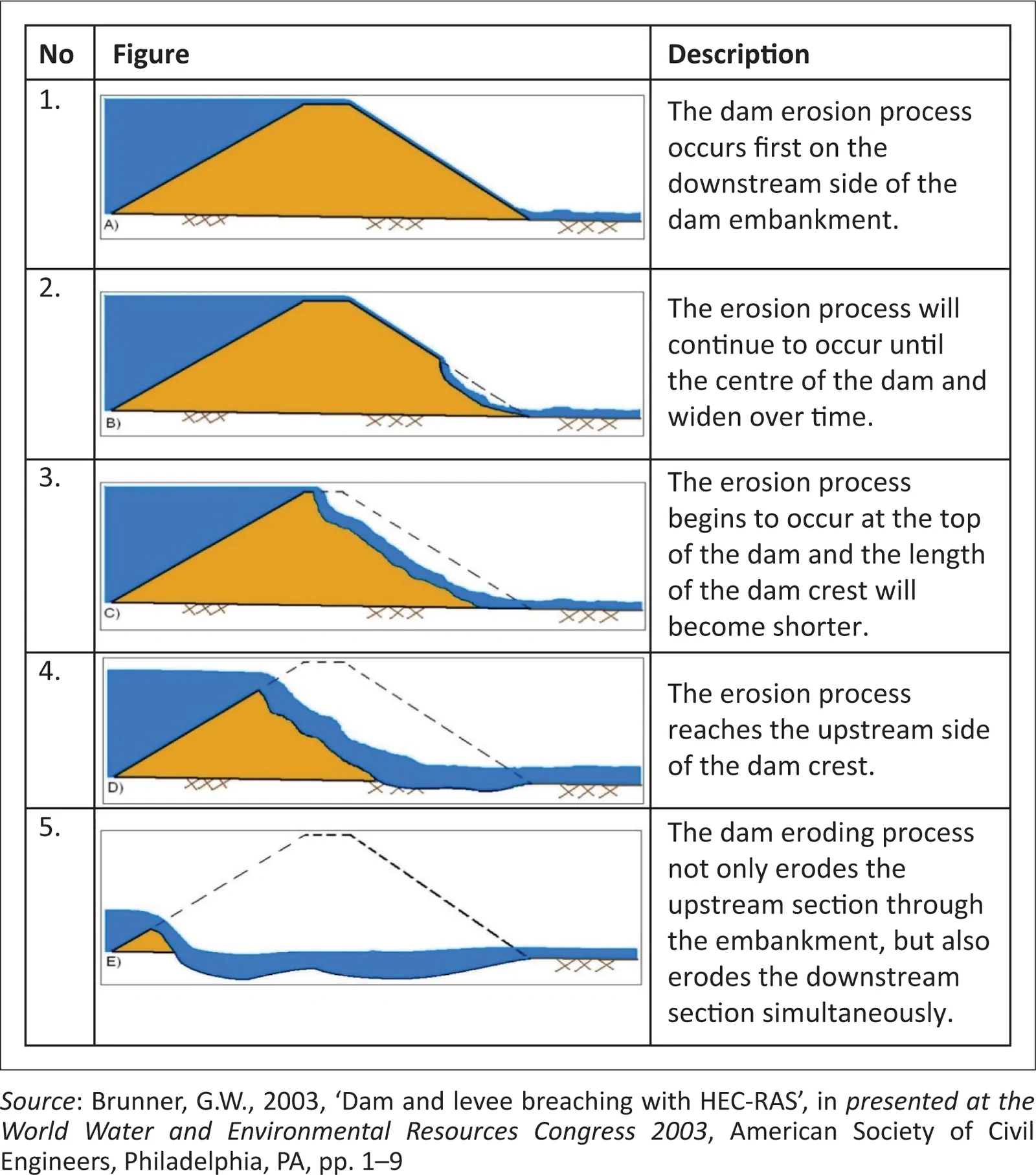
The overtopping process.

To provide a visual illustration of this phenomenon, the accompanying Figure 3 illustrates a dam failure caused by water overtopping.

### Simulation approach

The HEC-RAS 2D model simulated water propagation through the Keureuto River valley under a total breach scenario. The computational mesh resolution was 10 m × 10 m based on Digital Elevation Model Nasional Indonesia (DEMNAS) Geospatial data. Boundary conditions incorporated inflow hydrographs, outflow boundaries and roughness coefficients for land use types. The simulation generated time-series outputs for flood depth, velocity and extent, which were used to map hazard zones for risk mitigation.

A mesh resolution of 10 m × 10 m was selected to achieve an optimal balance between topographic representation and computational efficiency in the dam-break simulations. The topographic dataset was derived from DEMNAS, which has a spatial resolution of 10 m; therefore, the computational mesh was aligned with the native digital elevation models (DEM) resolution to minimise excessive interpolation and potential elevation distortion. This mesh resolution was considered sufficient to represent the morphology of the main river channels and floodplains, capture topographic variations within residential and agricultural areas and simulate stable flood-wave propagation.

Coarser meshes (> 20 m) tend to smooth critical topographic features, thereby reducing the accuracy of inundation depth and flood extent estimates. In contrast, finer meshes (< 5 m) substantially increase computational time and memory demand without providing a commensurate improvement in simulation accuracy relative to the quality of the available DEM data.

A sensitivity analysis was performed using mesh resolutions of 5 m, 10 m and 20 m. The results indicated that the difference in predicted inundation area between the 10 m and 5 m meshes was less than 5%, whereas the computational time for the 5 m mesh increased by more than twofold. Accordingly, the 10 m mesh resolution was adopted as the optimal configuration for the two-dimensional hydraulic simulations.

### Ethical considerations

This article followed all ethical standards for research without direct contact with human or animal subjects.

## Results

Simulation results show that dam failure would produce a flood wave with a maximum depth of 19.577 m and an inundation area of about 49.91 km^2^, posing severe risks to downstream settlements, infrastructure, agriculture and public facilities. Flood depths exceeding 3 m in residential areas indicate a high potential for structural damage and fatalities without timely evacuation.

Flood arrival time is critical for evacuation planning, as several downstream areas may be affected shortly after the dam failure. Therefore, automated early warning systems integrated with sensors, sirens, mobile alerts and centralised disaster management are recommended for rapid information dissemination.

The findings are important for evacuation planning and emergency response. Areas with the earliest flood arrival should be prioritised for evacuation, while critical infrastructure within the inundation zone should be assessed for reinforcement or relocation.

The simulation results support local governments and dam authorities in developing realistic emergency response scenarios, including estimating affected populations, planning logistics and shelters, prioritising aid distribution and preparing comprehensive emergency action plans (EAPs). From a long-term mitigation perspective, this study emphasises continuous dam safety monitoring through routine inspections, deformation monitoring and spillway evaluations. Dam-break scenarios should also be regularly updated to address increasing extreme rainfall risks linked to climate change.

Compared with previous dam-break studies in Indonesia and abroad, the results show similar hydraulic characteristics, including rapid downstream flood propagation, concentrated flow in valleys and extensive inundation in low-lying areas.

### Flood distribution and depth

The sediment breach parameters in HEC-RAS include breach geometry, failure timing and elevation, reservoir water level, and side-slope gradient. The dam material coefficient typically ranges from 0.5 to 0.6, whereas the overflow coefficient ranges from 2.6 to 3.0 (Li et al., 2021; Al-Iraqi, 2025).

**FIGURE 4:**
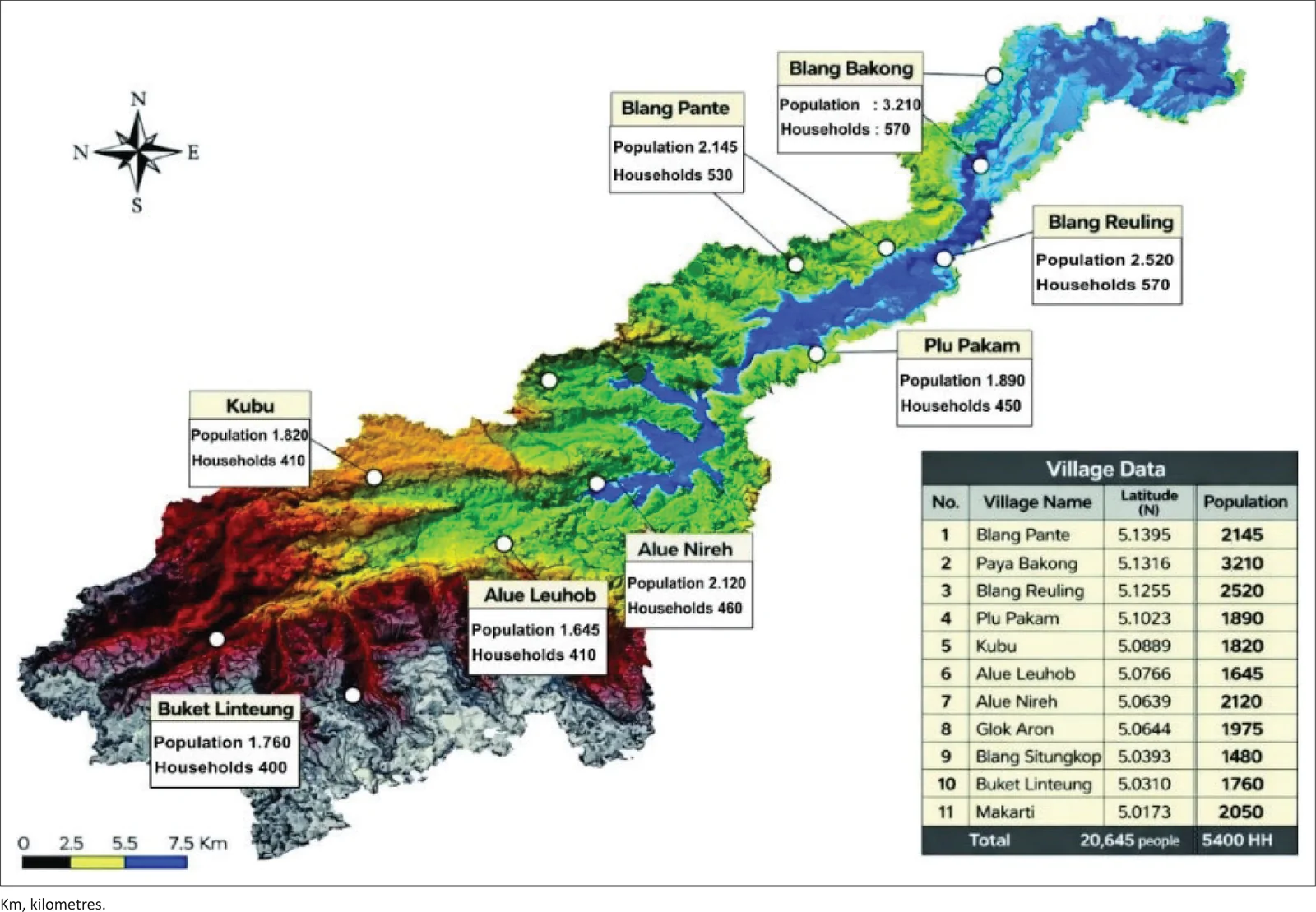
Flood distribution map from Keureuto Dam failure simulation.

The HEC-RAS simulation revealed extensive inundation downstream of the Keureuto Dam (Figure 4). Floodwaters spread rapidly through low-lying villages in Matang Kuli, Paya Bakong, Pirak Timu and Tanah Luas sub-districts. The simulation results illustrating the flood distribution caused by the Keureuto Dam collapse under overtopping conditions are presented in Figure 4.

**FIGURE 5:**
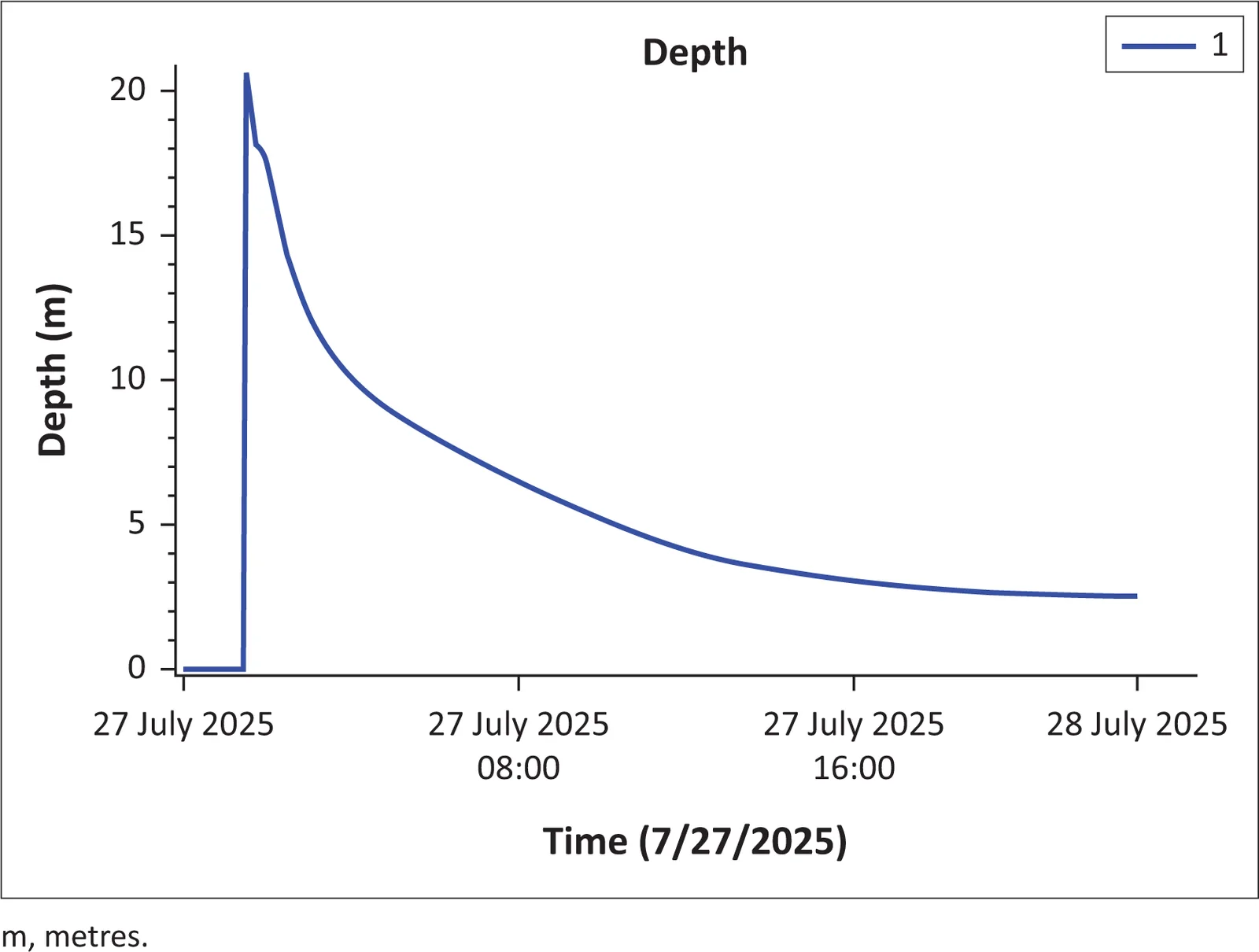
Time–depth graph of downstream water surface after dam collapse.

The maximum flood depth reached 19.577 m at 01:30, followed by 18.807 m at 01:35 and 18.032 m at 01:40. The inundation area was approximately 49.91 km^2^, covering 84 villages. Figure 5 presents the temporal variation in water depth.

Initially, flood depth rose sharply, indicating rapid dam breach erosion, then declined as discharge stabilised. Within about 3 h, depths reduced to below 2 m, and by noon, the floodwater receded to less than 1 m. This pattern is crucial for disaster preparedness, as it provides the time window available for evacuation and emergency actions in downstream communities (Mehta, Weeks & Tyquin 2020).

### Flow velocity and dynamics

The dam-break simulation shows high flow velocities near the breach and along the main river channel, causing rapid flood-wave propagation and increasing the risk of severe erosion and damage to bridges, roads and residential buildings.

High flow velocities and deep inundation greatly increase hydrodynamic forces on structures and reduce evacuation time. Areas near the river corridor experience the highest depths and velocities, indicating very high hazard intensity.

The velocity distribution is influenced by topography and land-cover roughness. Flow slows in vegetated and agricultural areas due to higher Manning’s coefficients, while the main river channel maintains higher velocities because of lower hydraulic resistance.

From a disaster management perspective, flow velocity strongly affects human safety, vehicle stability and structural vulnerability. High velocities combined with deep inundation pose major risks to downstream settlements and infrastructure, highlighting the need for early warning systems, evacuation planning and flood-resilient infrastructure.

**FIGURE 6:**
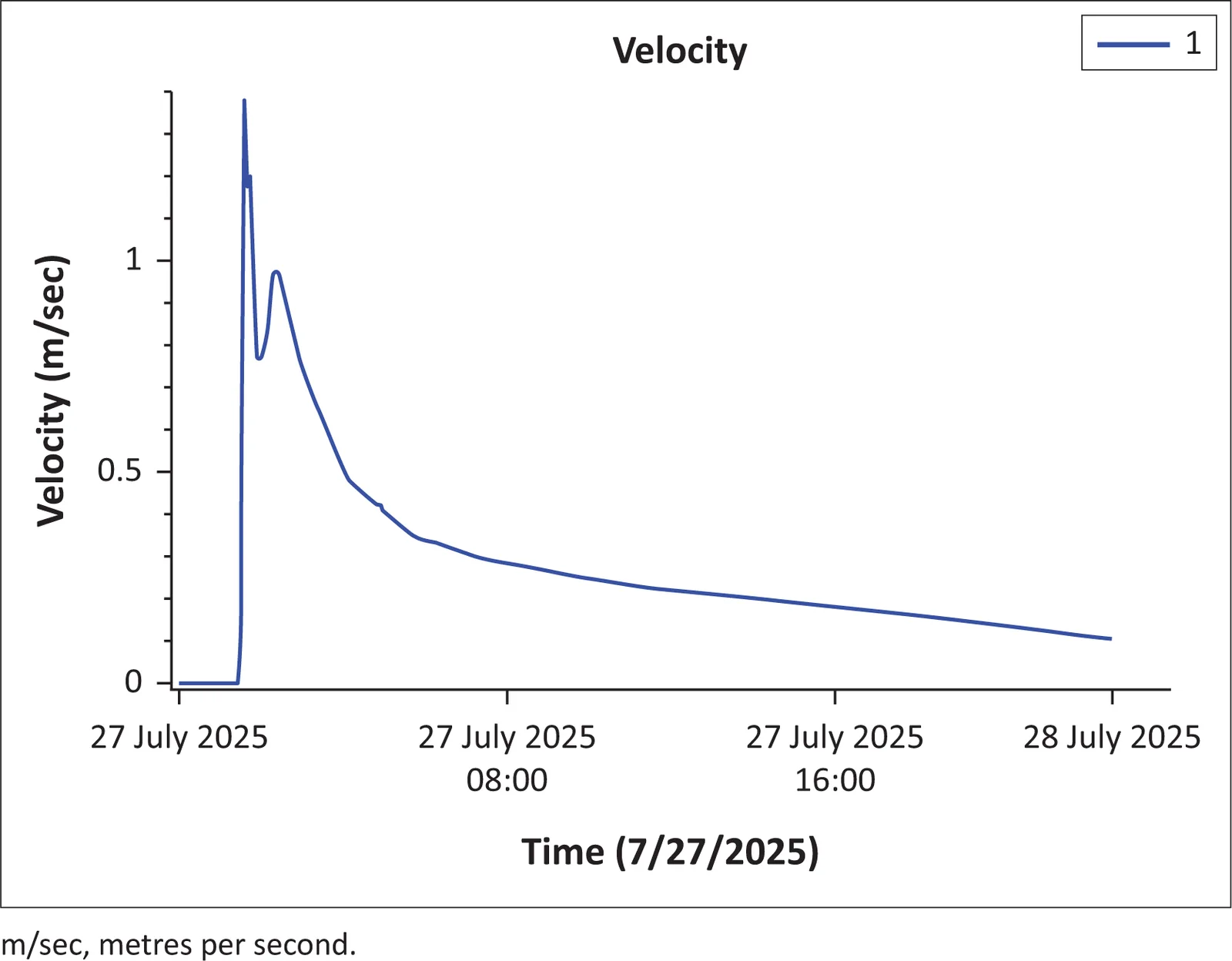
Flood velocity variation after dam collapse.

Figure 6 shows the flow velocity evolution after the breach. Peak velocity reached 1.389 m/s at 00:50, gradually decreasing below 1 m/s after 1:10.

These results indicate that the most dangerous period occurs within the first 90 min of dam failure. Such quantitative insights enable authorities to define critical response times and prioritise early-warning dissemination within that window. Velocity data also assist in assessing structural vulnerability, since buildings exposed to flows above 1 m/s face a high risk of damage or collapse (Elsanadedy et al. 2023; Song et al. 2024).

### Hydrological modelling for risk mitigation

Hydrological modelling is a representation of hydrological cycle processes used to simulate the movement of water within a watershed. This model includes processes such as precipitation, infiltration, evapotranspiration, surface runoff and river flow. In the context of risk mitigation, hydrological modelling functions to predict extreme events such as floods and droughts, identify disaster-prone areas and support water infrastructure planning and spatial planning.

According to Ven Te Chow et al. (1988), hydrological models are essential tools for understanding hydrological responses to changes in environmental conditions and land use. Hydrological modelling plays a crucial role in disaster risk mitigation, particularly in flood mitigation, where models are used to simulate peak discharge, flood forecasting and the planning of dams and drainage systems. In drought mitigation, hydrological modelling is applied for water balance analysis, prediction of water deficits and irrigation planning. For watershed management, it is used to evaluate land use changes and analyse the impact of urbanisation on runoff.

Overall, hydrological modelling is a highly important tool in disaster risk mitigation, especially for floods and droughts. With its ability to simulate various scenarios, it supports better decision-making in infrastructure planning, water resources management and disaster risk reduction.

### Model validation and limitations

Although direct field validation is limited due to the dam’s recent construction, simulated peak discharges and floodplain extents were compared with historical floods in the region and found to be consistent with topographic conditions. Nevertheless, model accuracy may be affected by DEM resolution, simplified breach geometry and absence of sediment transport coupling (Harada et al. 2019; Tazaki, Harada & Gotoh 2022).

Despite these constraints, the integrated hydrological–hydraulic modelling approach provides a reliable framework for risk assessment and scenario planning. Future work should include multi-scenario simulations (e.g. piping, seismic failure) and integration with socioeconomic exposure data to support holistic risk reduction strategies.

To improve the comprehensiveness of the dam-break assessment, a piping failure scenario should be included alongside overtopping, since internal erosion is a common cause of embankment dam failure. Incorporating this scenario would allow comparison of flood characteristics under different failure mechanisms and strengthen hazard assessment and emergency planning.

Alternatively, if the piping scenario is excluded, a stronger technical justification should be explicitly stated. The exclusion can be justified by the structural and geotechnical characteristics of the Keureuto Dam, which indicate a relatively low susceptibility to internal erosion. The dam incorporates an impervious core, graded filter layers and internal drainage systems designed according to modern embankment dam safety standards to prevent seepage-induced erosion. In addition, geotechnical monitoring records and seepage evaluations conducted during the operational period have not indicated abnormal pore-water pressure, excessive seepage discharge or signs of internal material transport that could trigger piping initiation. Therefore, the present study prioritises the overtopping failure mechanism because extreme hydrological loading associated with the PMF represents the most critical and credible worst-case scenario for the Keureuto Dam. Nevertheless, future studies are recommended to incorporate multi-scenario failure analyses, including piping and combined failure mechanisms, to reduce uncertainty and improve dam safety risk evaluation.

### Recommendations

The following recommendations are proposed based on the findings of this study:

- **Early Warning and Real-Time Monitoring:** Install an automated telemetry system that integrates rainfall sensors, reservoir water level gauges and prediction modules based on HEC-RAS to trigger early warnings. Alerts should reach at-risk communities within 30 min after abnormal readings are detected.
- **Community Preparedness and Training:** Conduct regular evacuation drills and awareness programmes in the 84 affected villages to ensure a rapid and well-organised response during dam-related emergencies. Disaster education should emphasise flood arrival times and safe evacuation corridors derived from model outputs.
- **Land Use and Spatial Planning:** Utilise inundation and flood velocity maps to guide regional zoning regulations. Development and settlement expansion should be prohibited in high-risk floodplain zones identified by the model.
- **Integration with Multi-Hazard Risk Frameworks:** Future studies should incorporate potential pipe failure, seismic events or cascading effects and evaluate compound hazard scenarios. Integrating hydrological modelling with socioeconomic vulnerability mapping will produce more holistic risk assessments.
- **Model Improvement and Calibration:** Future modelling efforts should integrate sediment transport and structural deformation modules, use higher-resolution DEM and validate results through field surveys and sensor data after dam operations commence.

By implementing these recommendations, government agencies such as the River Basin Agency of Sumatra I and the Aceh Regional Disaster Management Agency (BPBD) can enhance flood preparedness and reduce potential loss of life and property in future dam-related disasters.

## Conclusion

This study applied hydrological and hydraulic modelling to assess and mitigate disaster risks associated with the potential failure of the Keureuto Dam in North Aceh, Indonesia. Using the HEC-RAS 5.0.7 two-dimensional simulation framework and hydrological inputs derived from statistical frequency analyses, the study demonstrated the capability of modelling tools to quantify hazard parameters and inform proactive disaster management strategies. Key findings are as follows: The PMF was estimated at 5082.07 m^3^/s, serving as the design inflow for the dam breach scenario. Under overtopping conditions, the model simulated a maximum flood depth of 19.577 m, a peak velocity of 1.387 m/s and an inundation area of approximately 49.91 km^2^. The flood wave affected 84 villages across four sub-districts, Matang Kuli, Paya Bakong, Pirak Timu and Tanah Luas, indicating substantial downstream exposure. The most critical period for emergency response was within the first 90 min following dam breach initiation, when flow velocities exceeded 1 m/s. Beyond hydrodynamic quantification, the results underscore the pivotal role of hydrological modelling in disaster risk mitigation. The generated flood hazard maps and hydrographs provide essential inputs for: developing emergency action plans (EAPs), optimising early warning system response times and designing community-based preparedness and evacuation programmes. By integrating modelling outcomes into policy and planning, regional authorities can transition from reactive disaster response to evidence-based, anticipatory risk management. This research thus contributes to strengthening local resilience, aligning with the Sendai Framework for Disaster Risk Reduction’s priority on understanding disaster risk through scientific and technological innovation.
